# Oral cancer in young adults: incidence, risk factors, prognosis, and molecular biomarkers

**DOI:** 10.3389/fonc.2024.1452909

**Published:** 2024-09-20

**Authors:** Deborah Lenoci, Elisa Moresco, Stefano Cavalieri, Cristiana Bergamini, Erica Torchia, Laura Botta, Silvana Canevari, Annalisa Trama, Lisa Licitra, Loris De Cecco

**Affiliations:** ^1^ Integrated Biology of Rare Tumors Unit, Department of Research, Fondazione IRCCS Istituto Nazionale dei Tumori, Milan, Italy; ^2^ Head and Neck Medical Oncology Department, Fondazione IRCCS Istituto Nazionale dei Tumori, Milan, Italy; ^3^ Department of Oncology and Hemato-Oncology, University of Milan, Milan, Italy; ^4^ Evaluative Epidemiology Unit, Department of Epidemiology and Data Science, Fondazione IRCCS Istituto Nazionale dei Tumori, Milan, Italy; ^5^ Retired, Milan, Italy

**Keywords:** oral cavity squamous cell carcinoma, young adult, incidence, risk factors, outcome, molecular data

## Abstract

Oral cavity squamous cell carcinoma (OCSCC) predominantly affects the tongue and the floor of the mouth, primarily in patients over 50 years of age. Incidence and mortality rates vary significantly worldwide, influenced by geographic areas and demographic characteristics. Epidemiological studies revealed an increase in incidence of OCSCC among young adults (YA) <44 years old. This narrative review, provides updated information on the incidence, risk factors, and prognosis of YA-OCSCC using data published from 2018 to 2023 from different geographic locations. The studies indicate that the incidence of YA-OCSCC in Asia is approximately twice that in the US and that the incidence is strongly linked to risk factors such as betel quid chewing, tobacco use, and high alcohol consumption. The prognosis for YA-OCSCC, compared to that in older patients, shows similar or better overall survival, even in cases with relapses, but worse 5-year disease-free survival, despite receiving similar treatments. Consequently, a concerted effort is crucial to raise awareness about the cessation of tobacco and areca nut use, alcohol control, and the promotion of healthy lifestyle behaviors. Recent molecular data on YA-OCSCC suggests a potential profile characterized by epidermal growth factor receptor overexpression, low tumor mutation burden and an attenuated immune response. Upon confirmation in larger cohorts of YA-OCSCC patients from different geographical areas, the validated markers could aid in selecting tailored treatments.

## 1 Introduction

**Figure 1:**
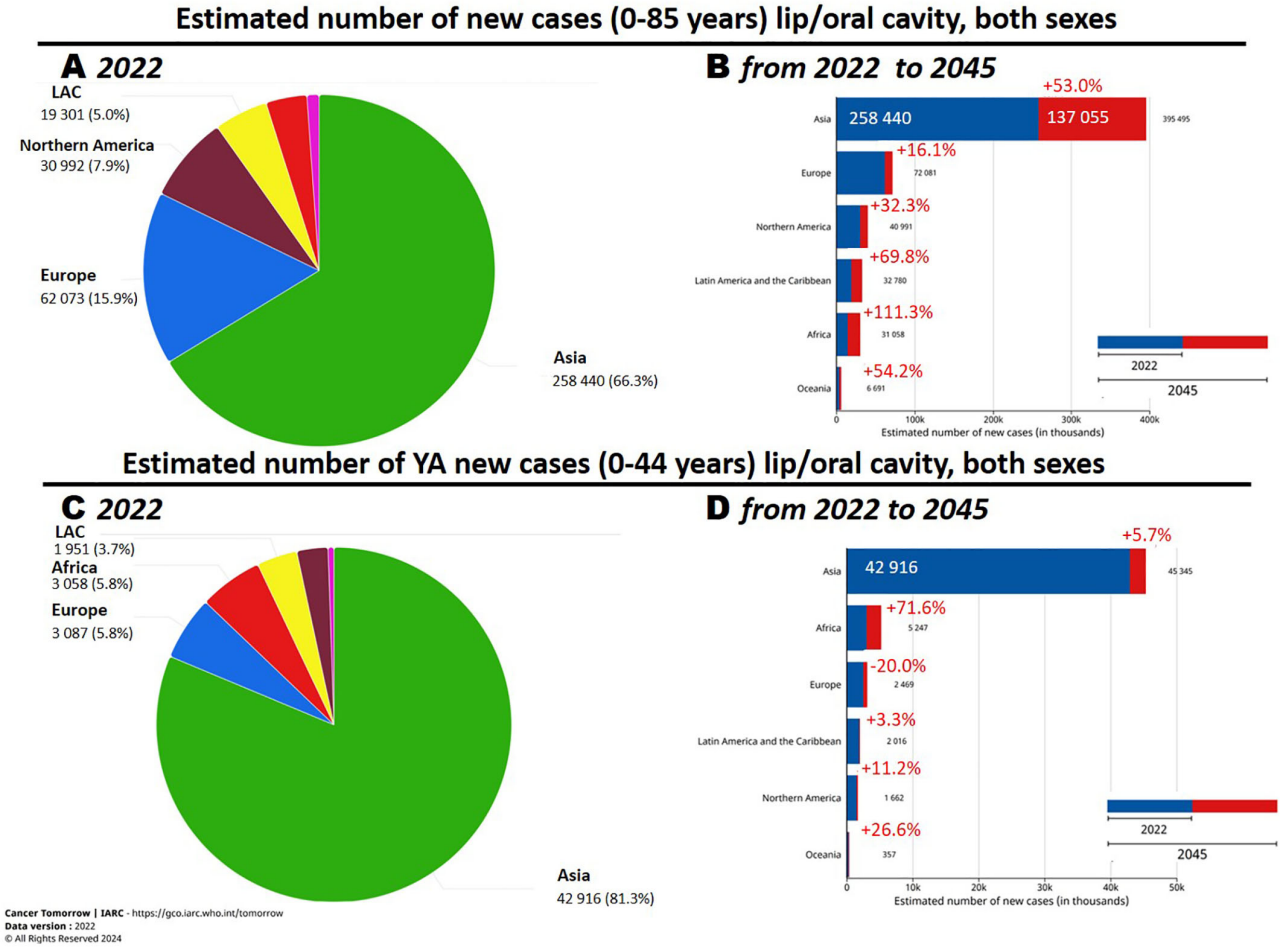
**(A, C)** Number of new oral cavity cancer cases in 2022 and **(B, D)** estimated changes in 2045. Reprinted with permission from the online data visualization tools for exploring the global cancer burden provided by the IARC (International Agency for Research on Cancer) Global Cancer Observatory. Figures “Cancer Today. Absolute numbers, Incidence, Both sexes, in 2022. Lip, oral cavity. Continents”, “Cancer Today. Absolute numbers, Incidence, Both sexes, age [0-44], in 2022. Lip, oral cavity. Continents”, “Cancer Today. Age-Standardized Rate (World) per 100 000, Incidence, Both sexes, in 2022. Lip, oral cavity. Continents”, “Cancer Today. Age-Standardized Rate (World) per 100 000, Incidence, Both sexes, age [0-44], in 2022. Lip, oral cavity. Continents” Respective URLs (accessed on 17th June 2024): https://gco.iarc.fr/today/en/dataviz/pie?mode=population&group_populations=0&cancers=1 https://gco.iarc.fr/today/en/dataviz/pie?mode=population&group_populations=0&cancers=1 &age_end=8&populations=903_904_905_908_909_935 https://gco.iarc.fr/today/en/dataviz/bars?mode=population&cancers=1 https://gco.iarc.fr/today/en/dataviz/bars?mode=population&cancers=1&age_end=8 Copyright 2024. Permission from IARC obtained on 17th June 2024.

Oral cavity squamous cell carcinoma (OCSCC) is the predominant histotype among oral cavity malignant tumors, comprising over 90% of cases. GLOBOCAN estimates (2020) (1) indicate that there are approximately 380,000 new OCSCC cases worldwide, with an age-standardized mortality rate of 2.8 per 100,000 in men and 1.0 per 100,000 in women. The Global Cancer Observatory (GCO) (2), an interactive web-based platform maintained by the International Agency for Research on Cancer (IARC), reports that, among the six continents, Asia has the highest incidence of oral cancer (including lip and tongue) (65.8% of all cases), followed by Europe (17.3%) and North America (7.3%) (**Figure 1A**). Furthermore, GCO (2), offers projections for new cases between 2022 and 2045 through the “Cancer tomorrow” link (https://gco.iarc.fr/tomorrow). These projections suggest that the greatest increase in new cases will occur in Africa, with the smallest increase in Europe (**Figure 1B**). Notably, incidence and mortality rates vary significantly worldwide, influenced by geographic areas and demographic characteristics. Additionally, over 70% of OCSCC deaths occur in countries with a low or medium human development index (3).

OCSCC involves the tongue and the mouth floor, predominantly in patients >50 years of age, with a male-to-female ratio of about 2:1. However, epidemiological studies have observed a notable incidence of cases, especially tongue cancer, in young adults (YA) <44 years old. Although the estimates found in GCO are for tumors of the oral cavity including lips, in the age group 0-44 years the incidence of tumors of the lips is much lower than that of the oral cavities, less than 10% of the total, so it can be assumed that for this specific age group the estimates provided by GCO are a good approximation for OCSCC tumors (http://rarecarenet.istitutotumori.mi.it/analysis.php).

In 2020 GCO identified that approximately 17% of OCSCC were diagnosed in young–adult patients (**Figure 1C**) and “Cancer tomorrow” link estimates that the number of new OCSCC cases from 2020 and 2040 will have the higher increase in Africa and a relevant decrease in Europe (**Figure 1D**).Before 2018, we found only one paper analyzing big population data, all the others were hospital-based and the analyzed small cohort. The paper analyzed a comprehensive screening of the Taiwanese general population aged >18 years between 2004 and 2009 (> 18 million individuals). From this cohort > 4 million persons were identified as high-risk population and included in the screening. 4,110 OCSCC were identified and 42% of them were in the YA age range (4). A recent epidemiological study, based on a population cancer registry in Taiwan, depicting long-term trends of OCSCC from 1980 to 2019, identified Taiwan as having the highest incidence worldwide (5). Indeed, between 1980–1984 and 2015–2019, the age-standardized incidence rates increased from 4.19 to 27.19 per 100,000 in men and from 1.16 to 2.8 per 100,000 in women.

At the end of 2018, a seminal review aimed at analyzing early-onset OCSCC within the USA summarized the literature on risk factors, outcomes, and molecular analyses (6). Although this review referenced numerous studies, the vast majority were published before 2015, focused on a single geographic area, and involved limited patient cohorts. Furthermore no data about molecular genetic aspects were reported. Over the past seven years, an increasing body of evidence has emerged from various geographic areas. Therefore, the objectives of this “narrative review” are: i)present and discuss data published from 2018 to 2023; ii) summarize the new data about genetic predisposition, viral infections, and the preliminary identification of molecular data as potential risk factors or biomarkers.

## 2 Search strategy and selection criteria

A literature search was conducted using the PubMed database to identify studies pertinent to the epidemiology, risk factors, prognosis, and molecular data in YA-OCSCC patients. The search spanned from January 2018 to November 2023 and letters, editorials, study protocols, case reports, short communications, and non-English articles were excluded. Independent researchers, three in molecular biology and two in the clinical management of patients with head and neck squamous cell carcinoma (HNSCC), evaluated the articles for quality and thematic relevance. Additionally, the references of all included papers were reviewed to identify further studies of interest. The final selection was revised by two senior researchers.

## 3 Incidence of YA-OCSCC and associated risk factors in different countries

OCSCC constitutes a major global health challenge (1), with its incidence notably higher in certain geographic regions. India is regarded as the global epicenter of OCSCC (7), however, a limitation in analyzing the Indian context is that cancer registries encompass merely 10% of the population, predominantly in urban areas, while 72% of the population resides in rural settings. Consequently, the incidence rates reported may be an underestimation of the true figures (7).

Five recent studies on YA-OCSCC incidence, with a high number of included cases (8–12) are reported in **Table 1**. Two studies (8, 9) reported that the incidence of YA-OCSCC in North America was around 10%. According to “Tracking changes in the age distribution of head and neck cancer in the United States from 1975 to 2016” (data from the SEER-NCI program), the mean age at HNSCC diagnosis has increased over the last 40 years, with YA-OCSCC being the only HNSCC subset to demonstrate a significant increase in proportion, likely due to a rise in diagnoses among young women (15). We also included in **Table 1** two individual studies: the first from two referral centers in India (13) and the second discussing risk factors, prognosis, and treatment strategies for YA-OCSCC in patients aged newborn to 20 years (14).

**Table 1:** YA proportion in OCSCC.

Author-year [ref] DOI	Years	Data source Geographical area	YA-OCSCC < 39–44 y (N)	Overall OCSCC N		YA %
Data from the largest analyses						
Oliver J. 2019 (8) 10.1002/hed.25772	2004-2015	National Cancer Database North America	2,566		22,930	10%
Mohideen K. 2019 (11) 10.4103/jomfp.JOMFP_118_19	2014–2019	Meta-analysis (24 studies: China, 5; USA, 4; Brazil, 3; Pakistan, India, Korea, 2; 9 single studies from different countries)	4,563		42,295	10%
Lee DS. 2021 (9) 10.1002/lary.29260	1988–2019	Publications, Meta-analysis North America (72%) Asia (21%) Others (7%)	2,238		23,382	10%
Ferreira E. 2022 (10) 10.1007/s12105-022-01441-w	1998–2018	Meta-analysis different parts of the globe	626		10,727	6%
Battistella EA. 2022 (12) 10.1007/s00784-022-04719-z	1977– 2021	Publications on OCSCC patients reporting tobacco and alcohol consumption*	2439		13,393	15%
Data from single studies						
Abdulla R. 2018 (13) 10.4103/jomfp.JOMFP_16_18	1996–2012	2 referral centers South Western coast India	86		420	20%
Best DL. 2021 (14) 10.1016/j.joms.2020.12.018	1894–2020	64 studies with OSCC with age at presentation newborn- 20 years	108 YA-OCSCC			

The 38 included studies originated from eighteen countries: Australia, Brazil, Israel, North America (Canada, USA), Europe (France, Germany, Italy, Spain, Ireland), and Asia (China, India, Singapore, South Korea, Sri Lanka, Japan, Taiwan, Thailand).

The most recent meta-analysis on YA-OCSCC (10), including different continents, did not identify an increase in YA-OCSCC incidence within the analyzed timeframe (1998–2018). However, the YA-OCSCC trend in incidence in the population under study (South America, China, Europe, India, and South Africa) was 6%, while in India, it was more than double at 13%. The single study (13) reporting a 20% prevalence of YA-OCSCC appears to confirm its higher prevalence in Asia compared to that of older patients. The established risk factors for YA-OCSCC include tobacco use, high alcohol consumption, and betel quid chewing (16). In North America and Europe, alcohol consumption and tobacco smoking are widespread and may persist throughout life. In South-Central Asia, tobacco smoking and betel quid chewing are more common practices.

**Figure 2:**
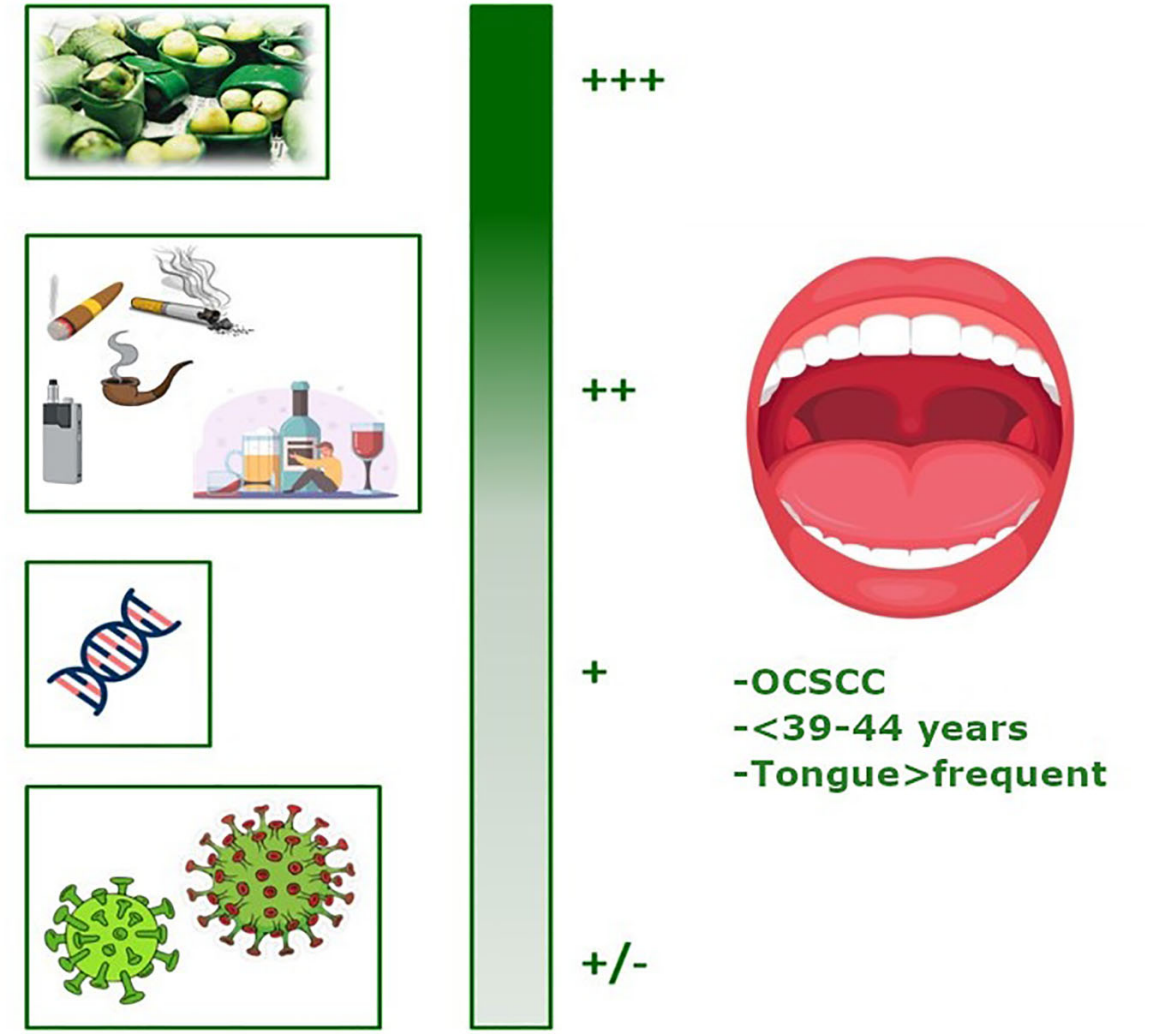
Graphical representation of the factors involved in the development of YA-OCSCC (ranked by probability). Created with BioRender.com.

**Figure 2** summarizes the factors involved in the development of YA-OCSCC, categorized according to their likelihood of causing the tumor. Approximately 600 million people chew betel quid in India and Southeast Asia (17). Betel quid typically contains areca nut, betel leaf, catechu, slaked lime, and often tobacco (18). The areca nut produces carcinogenic nitrosamines in the saliva of chewers, leading to oral preneoplastic disorders with a high likelihood of progressing to cancer (18). Consequently, IARC has classified it as a Group 1 carcinogen (19). Areca nut, the fruit of the Areca catechu palm, is widely cultivated in Asia and the Pacific Islands, where it is more popular (19). Betel nut chewing begins at a very young age in Micronesia, resulting in a high number of school children developing oral mucosal lesions (20). Efforts by the World Health Organization to control betel nut chewing face challenges due to its deep cultural roots, including religious significance in some parts of Southeast Asia and India (17).

The new evidences confirm that the *Betel quid chewing* represents the factor with the highest probability of causing cancer (**Figure 2**: +++).

Data about *tobacco smoking and alcohol consumption* (**Figure 2**:++) indicated that are still the most prominent causes of cancer worldwide and are considered significant for OCSCC occurrence, and, as evidenced by studies published in the recent years, the comparison of the proportion of individuals reporting tobacco and alcohol consumption showed that these habits were more prevalent in the older group (48.4% and 45.8%, respectively) than in the younger group (39.5% and 30.9%, respectively). Schantz et al. (21) reported that the proportion of YA patients with a history of tobacco use (cigarette smoking or tobacco chewing) decreased from 64% to 35% between 1944 and 1984. A similar trend was observed in patients who were drinkers.

During the same period, the popularity of smokeless tobacco products, such as electronic cigarettes (ECs) or vaping, has increased worldwide, driven by the belief that they are safer alternatives to traditional smoking. These studies found that although EC users have a lower cancer incidence than do conventional cigarette smokers, their cancer burden remains twice that of nonsmokers (22). Campbell et al. (23) conducted an extensive study in US referral centers evaluating the risk factors associated with YA-OCSCC. This was the first study to report an association between YA-OCSCC and EC use in the US. Despite the absence of a control group, the proportion of EC use among YA-OCSCC patients (12.4%) exceeded the national rates for similar age groups (5–6%), suggesting a potential role for EC in tumor development. Furthermore, a recent systematic review found that EC users tend to be YA males with higher education levels. This provides valuable insights for refining intervention strategies aimed at EC users to enhance the effectiveness of anti-smoking efforts (24).

The medium-low risk factors included *inherited genetic alterations* (**Figure 2**: +), such as those associated with Fanconi anemia (FA). FA, a rare hereditary disease typically autosomal recessive, is caused by mutations in one of 22 known FA-related genes (25). It is characterized by a deficiency in DNA damage repair, particularly inter-strand DNA crosslink repair, leading to bone marrow failure (26). FA also increases genomic instability in the epithelial cells of the head and neck region. Patients with FA have a significantly elevated risk of malignancy by age 40, with OCSCC being the most common solid tumor observed (the estimated incidence of OCSCC is higher in patients with FA than in the general population). Moreover, as the life expectancy of patients with FA improves, the incidence of OCSCC (27) may rise over time.

Some YA-OCSCC patients without FA present with a monoallelic germline alteration in an FA gene and a second risk factor, such as APOBEC alteration (26). Additionally, other genetic predispositions are implicated in YA-OCSCC. For example, the rs6942067 GG genotype, a single nucleotide polymorphism upstream of the *DCBLD1* gene, was significantly more prevalent in the Cancer Genome Atlas HNSCC cohort among young, HPV-negative non-smokers with HNSCC (28). Furthermore, polymorphisms in the major histocompatibility complex class I-related chain A (MICA), which are critical for eliminating malignant tumors, have been associated with carcinogenesis in adolescents and YA with OCSCC, suggesting their potential as cancer markers in this age group (29). Studies on the impact of age at diagnosis indicate that YA-OCSCC risk increases with a positive family history of cancer (16). The International Head and Neck Cancer Epidemiology Consortium’s observation of this association (30) led Mahmood et al. (31) to conclude that genetic factors are likely to play key roles. Additionally, Best reported that OCSCC in individuals under 21 years of age, who lack a predisposing condition, likely represents a rare entity with an etiology that is not well understood (**Table 1**) (14).

Certain *virus* infections (**Figure 2**: +/-) may be potential risk factors. Over the past few decades, HPV infection has emerged as a risk factor for HNSCC, particularly in the oropharynx, most often originating from the tonsillar crypt epithelium. HPV oncoproteins inactivate specific proteins, disrupting cell cycle regulation, causing genetic instability, and increasing the proliferation of cancer cells, thereby promoting tumor development (16). Studies have indicated a higher incidence of HPV-related OCSCC in younger patients, typically 5–10 years younger than those who are HPV-negative and usually without established risk factors such as tobacco and alcohol consumption (32). Patients with HPV-related OCSCC exhibit distinct clinical and epidemiological features, including younger age at diagnosis, high-risk sexual behaviors, and improved survival rates, differentiating these cancers from HPV-negative ones (33). Hepatitis C virus (HCV) is another viral risk factor for OCSCC. Su et al. (34) studied the population in Taiwan, where both viral hepatitis and OCSCC are endemic, from 2000 to 2005. Comparing 21,199 adults with chronic HCV to 84,796 sex/age-matched subjects without viral hepatitis, they discovered that HCV-positive patients developed OCSCC at an earlier age (<50 years) than those in the viral hepatitis-free control group.

A recent systematic review and meta-analysis examining the epidemiology of OCSCC in non-smokers indicate a distinct profile compared to smokers with OCSCC, with a majority of cases being women (54–82%) and patients typically younger (<50 years) or older (>70 years) than their male counterparts at the time of diagnosis (35). The authors propose a multifactorial etiology while acknowledging that the current literature on the topic of non-smokers with OCSCC is limited, emphasizing the need for further study.

### 3.1 Outcome of YA-OCSCC

Whether age is an independent prognostic factor in OCSCC is debatable (**Table 2**). Recent retrospective studies have indicated that YA-OCSCC patients exhibit better outcomes than their older counterparts (23, 29, 48). Registry data, from Surveillance Epidemiology and End Result (SEER) (36) and one meta-analysis (39), consistently demonstrated superior overall survival (OS) in YA-OCSCC compared to older patients with OCSCC. However, two separate meta-analyses (9, 37), reported similar outcomes for both YA and older individuals. Moreover, a worse disease-free survival (DFS) (37, 39), and a higher risk of distant metastasis (39) have been observed in YA-OCSCC patients.

**Table 2:** Prognosis of YA-OCSCC.

Author-year [ref] DOI	Years	Data source Geographical area	YA-OCSCC < 39–44 y.	Overall OCSCC	Conclusions
Data from meta-analyses and largest analysis					
Mukdad L, 2018 (36) 10.1002/lary.27720	1973–2012	Surveillance, Epidemiology, and End Results (SEER) database North America	1,232	15,191	Better OS and DSS in: YA female compared to YA male
Lee DS. 2021 (9) 10.1002/lary.29260	1988–2019	Meta-analysis North America (72%) Asia (21%) Others (7%)	2,238	23,382	After definitive treatment, similar oncologic outcomes in YA and older OCSCC
Kaminagakura E, 2021 (37) 10.1002/hed.2694	1998–2019	Meta-analysis: 2 North America, 2 South America, 2 Europe, 5 Asia, 1 Oceania	449	1,168	5-year OS similar between groups, relapses and 5-year DFS worse in YA-OCSCC
Tagliabue M, 2021 (38) 10.1002/cam4.3795	1989–2019	Meta-analysis: 5 Europe, 9 USA, 9 Asia, 3 Israel, 2 Australia, 1 Saudi Arabia, 1 Canada, 1 Brazil	28,288^a^		YA-OCSCC has better OS but a higher relapse frequency
Panda S, 2022 (39) 10.3390/cancers14081886	1998–2021	Meta-analysis: 40% Asian populations, 60% European, Australian and North American populations	4,981	44,254	YA-OCSCC has better OS, worse DFS, higher frequency of recurrence and distant metastases
Data from single studies					
Campbell BR, 2019 (23)10.1002/hed.25650	2000–2017	Referral center US	113 (<50 y)	282	YA-OCSCC has better OS
Tani R, 2021 (29) 10.1016/j.oraloncology.2021.105256	1999–2017	Japan, MICA^b^ A5.1 homozygous genotype compared to other genotypes	12 A51.1 homozyg. (11<40 y)	374	YA-OCSCC with A51.1 genotype has better prognosis

^*^Tagliabue paper does not report the number of YA-OCSCC cases.

^§^MICA, Major Histocompatibility Complex Class I-Related Chain A.

These observations may be influenced by various confounding factors. Older individuals are often frailer than young adults; thus, it is possible that YA-OCSCC patients received more intensive treatment. Moreover, older individuals typically have more comorbidities, which suggests that competing causes of death could have affected the mortality rates reported in the studies. Additionally, while some research indicates that YA-OCSCC patients may experience poorer DFS, it is conceivable that their potentially better tolerance of salvage/palliative treatments may have favorably impacted their post-recurrence survival.

Based on the available literature and quality of the studies, no final conclusion can be drawn on the independent prognostic role of age for OCSCC. However, while conducting translational research studies on prognostic signatures for OCSCC patients, age should be regarded as a relevant clinical variable to be included in multivariable models.

### 3.2 Molecular data

The heterogeneity of HNSCC and OCSCC, along with the challenges in distinguishing hereditary factors from environmental ones, complicates the identification of a single set of genes involved in the diseases’ pathogenesis (16, 31). Furthermore, Mishra et al. (49) conducted an extensive literature survey and analysis to generate chromosome maps that specifically highlight OSCC-related mutations.

Recently, numerous experimental approaches have concentrated on the link between high-risk genes and YA-OCSCC. Kolegova et al. (16) recently summarized the specific genetic, epigenetic, transcriptomic, and proteomic alterations associated with YA-OCSCC. They emphasized the distinct tumor microenvironment and immune landscape in these patients. On these bases, we chose several recent studies that identified novel potential risk factors or biomarkers for early onset and/or prognosis (Table).

Notably, the geographical areas vary between studies on protein expression (Brazil and China) and those on genomics/transcriptomics alterations (North America, Australia, and Europe), allowing for only limited comparisons. However, where comparisons were feasible, Epidermal growth factor receptor (EGFR) protein over-expression (40) was corroborated by gene amplification (44). The tumor mutation burden (TMB) was observed to be lower in YA-OCSCC compared to older OCSCC patients in two distinct studies, one encompassing North America and Australia (44), and the other Asia and North America (46). Moreover, Parzefall et al., revealed that protein kinase C alpha (PRKCA), highly expressed in oral tongue squamous cell carcinoma (OTSCC) young patients without alcohol and smoking history, is associated with a poor prognosis and it can be used as a biomarker to predict high-risk patients (50).

In addition to the data reported in **Table 3** two recent studies have highlighted the role of the tumor immune environment in YA-OCSCC (22, 50). The first study (51), despite being limited to a small sample size (11 patients <44 years and five patients >44 years), provides detailed immunologic data that suggest older patients have significantly higher percentages of FOXP3 + T-cells and elevated expression levels of signatures related to anti-tumor immune activity. The second study (22), which reviewed molecular data from the literature, found that whole transcriptomic analysis of smokers, vapers, and non-users of tobacco products revealed dysregulation of immune-related and mitochondrial genes in both vapers and smokers. Similar to traditional smoking, vaping increases resistance to anti-cancer therapies, causes oral microbiome dysbiosis, and promotes suppression of the host immune system.

**Table 3:** New molecular data in YA-OCSCC.

Author-year [ref] DOI	Data source	Geographical area (N. cases)	YA-OCSCC < 39–44 y.	OCSCC > 39–44 y.	Conclusions
Immunohistochemistry					
Galvis MM. 2018 (40) 10.1111/jop.12601	Tissue microarray	Brazil	61	71	YA-OCSCC: EGFR and MMP-9 have significantly higher overexpression levels; correlation between c-ErbB-2 and SMA expression with lower DFS
Zhang B. 2019 (41) 10.2147/CMAR.S211847	Tissue sections 1995–2018	China	103	103 matched > 60 y	YA-OCSCC: neutrophil-to-lymphocyte ratio associated with better prognosis
Transcriptomics					
Maroun CA. 2021 (42) 10.1002/lary.28674	TCGA RNA dataset	USA	21	224	YA-OCSCC: attenuated immune response and lower frequency of immunogenic mutations
Transcriptomic and whole genome sequencing					
Gu X. 2018 (43) 10.1111/jop.12792	UNS, TCGA (RNA and genome seq)	Sweden (10) USA (115)	5 + 13	5 + 102	YA-OCSCC: better OS in low-CNV compared to high-CNV
Satgunaseelan L, 2021 (44) 10.3389/fonc.2021.750852	RNA and genome seq 2012–2018	Australia (17) US (9 + 2 TCGA)	26 <50 y.	11 >50 y.	YA-OCSCC: lower TMB and increased EGFR amplification
Genomics					
Bahethi RR, 2020 (45) 10.1177/2473974X20970181	Systematic review from 13 studies of non-smokers YA-OCSCC	(Europe: 3, USA: 6, Asia 4) Only 3 studies with comparison with older OCSCC	Tumors genetically sequenced or mutational profiles analyzed*		*TP53* is the most commonly evaluated gene; no difference in mutations between non-smokers YA-OCSCC and older OCSCC
Campbell RB. 2021 (46) 10.1002/cncr.33309	Sequencing Consortium Oral Tongue Cancer (TCGA +7 Genomic studies)^§^	US 62% India 23% Singapore 12% Sweden 3%	107	120	YA-OCSCC: smaller TMB not explained by differences in tobacco use
Satgunaseelan L, 2022 (47) 10.1002/gcc.23076	Whole genome sequencing 2012–2018	Australia (28) TCGA US (9)	37 <50 y	–	High frequency of WG duplication in YA-OCSCC is associated with adverse pathologic characteristics and worse outcomes

^*^See data of the specific study data for the number of cases and to **Table 1** in the paper for the list of analyzed genes.

^§^See Study-specific inclusion criteria

## 4 Conclusions

The update of the YA-OCSCC literature and the evaluation of IARC data about the YA-OCSCC incidence enabled to better define the burden of this disease in different geographic regions and to suggest its association with the major risk factors (betel quid chewing, tobacco use, and high alcohol consumption).

Based on the “Cancer tomorrow” link [https://gco.iarc.fr/tomorrow], the estimated number of new YA-OCSCC cases between 2020 and 2040 is expected to increase in Africa and decrease in Europe. Moreover, concerted efforts by healthcare professionals, policymakers, and the community are expected to enhance awareness among YAs through education on tobacco, including also e-cigarette and areca nut use cessation, alcohol control, and the promotion of a healthy lifestyle (3, 12, 37). For cases not associated with lifestyle factors, new genetic alterations/predispositions in YA-OCSCC have been identified as potential risk factors. However, due to the limited number of cases, further studies involving larger cohorts and high-throughput genomic approaches are necessary.

Recent YA-OCSCC prognostic studies, which compared outcomes with those of older patients with OCSCC, indicated similar or better OS, even in cases with relapse and a worse 5-year DFS. These findings suggest that young age alone may not warrant age-specific treatment selection beyond the standard of care. Thus, as per international guidelines (52), radical surgery should always be pursued, when feasible, with postoperative radiotherapy for loco-regionally advanced disease (i.e., stage III-Iva/b). The use of adjuvant concomitant chemoradiation is limited to cases with non-radical resections and/or lymph node involvement with extracapsular spread (53). Due to the relevant toxicities related to chemo-radiotherapy, concurrent adjuvant treatments are usually reserved to fit patients without major comorbidities. Given that older subjects are more frequently frail than younger individuals counterparts, we cannot exclude that elderly patients may have received less intensive treatments compared to YA-OCSCC. This potential confounder may have impacted on patient survival and, ultimately, on prognostic studies in general.

Molecular data studies associated with YA-OCSCC remain exploratory, having been performed in a limited number of cases. However, across different geographic areas the following profile has been proposed for YA-OCSCC: i) overexpression of EGFR; ii) low TMB; and iii) an attenuated immune response promoted by smoking, snuffing, vaping, and oral microbiome dysbiosis. When tested in a large cohort of YA-OCSCC patients, the identified markers could contribute to the selection of tailored treatments.

By analyzing epidemiologic cohorts and clinical studies, relevant etiological and molecular characteristics unique to YA-OCSCC have been identified. However, further research is needed to explore additional factors, such as the oral microbiome, sex, ethnicity, and various underlying genetic elements, that may influence the development and outcomes in YA-OCSCC patients.
